# Genome wide analysis of the evolution of Senecavirus A from swine clinical material and assembly yard environmental samples

**DOI:** 10.1371/journal.pone.0176964

**Published:** 2017-05-05

**Authors:** Wanhong Xu, Kate Hole, Melissa Goolia, Bradley Pickering, Tim Salo, Oliver Lung, Charles Nfon

**Affiliations:** National Centre for Foreign Animal Disease (NCFAD), Canadian Food Inspection Agency, Winnipeg, MB, Canada; Oklahoma State University, UNITED STATES

## Abstract

Senecavirus A (SVA), previously known as Seneca Valley virus, was first isolated in the United States in 2002. SVA was associated with porcine idiopathic vesicular disease in Canada and the USA in 2007 and 2012, respectively. Recent increase in SVA outbreaks resulting in neonatal mortality of piglets and/or vesicular lesions in sows in Brazil, the USA and Canada point to the necessity to study the pathogenicity and molecular epidemiology of the virus. Here, we report the analysis of the complete coding sequences of SVA from 2 clinical cases and 9 assembly yard environmental samples collected in 2015 in Canada, along with 22 previously released complete genomes in the GenBank. With this combined data set, the evolution of the SVA over a 12-month period in 2015/2016 was evaluated. These SVA isolates were characterized by a rapid accumulation of genetic variations driven mainly by a high nucleotide substitution rate and purifying selection. The SVA sequences clustered in clearly defined geographical areas with reported cases of SVA infection. No transmission links were identified between assembly yards, suggesting that point source introductions may have occurred. In addition, 25 fixed non-synonymous mutations were identified across all analyzed strains when compared to the prototype SVA strain (SVV-001). This study highlights the importance of monitoring SVA mutations for their role in increased virulence and impact on SVA diagnostics.

## Introduction

Senecavirus A (SVA), also known as Seneca Valley Virus (SVA), belongs to the same family (*Picornaviridae)* as foot-and-mouth disease virus (FMDV) and swine vesicular disease virus (SVDV) but is the only species in the genus *Senecavirus* [1]. SVA was first isolated as a contaminant in cell culture media in 2002 before being found in pigs in the USA [1] and in 2007 SVA was detected in Manitoba pigs with vesicular lesions [2]. SVA was also suspected to be the cause of anorexia, lethargy, lameness and vesicular lesions in a boar in the USA in 2010 [3]. In late 2014 into 2015, a high number of SVA infections were observed in grower-finisher pigs and breeding herds in the US and Brazil, with SVA being the only virus isolated from some of the clinical cases in Brazil [4][5]. Similarly, pigs with ulcers in the nostrils and mouth accompanied by loss of appetite, lameness, plus mortalities of newborn piglets were observed in Guangdong Province in China in 2015. SVA was detected in pooled samples from these pigs [6]. Unlike in previous studies with SVV-001 which failed to induce disease in pigs [7], experimental studies with the 2015 SVA isolates have established a causal relationship between SVA and vesicular disease in pigs [8][9]. This apparently suggests an evolution of the virus into a more virulent phenotype.

Phylogenetic studies have largely contributed to a better understanding of the emergence, spread and evolution of many RNA viruses, for example, 2001 FMD outbreak in the UK [10], highly pathogenic avian influenza epidemics [11–13], and 2013–2015 Ebola virus epidemic in West Africa [14,15]. Picornaviruses possess some of the highest nucleotide substitution rates among RNA viruses, but the evolutionary potentials of SVA have not been examined in detail due to limited sequences available in the public database. Recent emergence of SVA in Brazil and reemergence in the US and Canada urged the need to investigate the evolutionary properties of this virus, which may help understand the epidemiology of SVA infection and the potential of this virus to become more virulent to its host.

In October 2015, one sow from Ontario and two sows from Manitoba Canada that had been exported to an establishment in Michigan, USA had vesicular lesions on their snouts. Lameness was also observed in 2 of the 3 sows. SVA was detected in clinical samples from these animals. These animals were traced back to the assembly yards and farms of origin in Ontario and Manitoba and additional samples were collected. Here, we describe the virus detection, genome sequencing and analysis for 2 SVA isolates from these clinical cases in Ontario, Canada in 2015. In addition, we provide an analysis of complete coding- region of SVA genomes of 9 isolates from assembly yard environmental samples from Manitoba, Canada. We compared these genomes to 22 complete SVA genomes in the GenBank: 16 genomes from the United States, 3 genomes from Brazil and 3 genomes from China. We used this combined data set to understand levels of genetic diversity within Canada and between countries as well as molecular evolution of SVA, including nucleotide substitution rate and selection pressure in 2015/2016.

## Materials and methods

### Ethics statement

Samples used in this study were collected as part of a disease investigation by veterinarians licensed to practise veterinary medicine in Canada by the Canadian Veterinary Medical Association. The National Animal Care Farm Council’s Code of Practice for the Care and Handling of Pigs was followed. No institutional animal use authorization was required because no laboratory animal experimental work was performed.

### Samples

Tissue scrapings from pigs with lesions were placed in 5 mL of transport medium composed of 0.08 M phosphate buffer, pH 7.2 and 50% glycerol (VDTM) [16]. For environmental samples, a pair of cotton swabs were moistened in glycerol-free transport medium, gentle rubbed on selected surfaces and then submerged into 5 mL of the same transport medium. Swabbed surfaces are shown in Table 1. Samples were transported to the laboratory on ice packs and a secure container [16].

**Table 1:** Assembly yards, surfaces sampled and SVA detection by real-time reverse transcription polymerase chain reaction.

	Assembly yard A in Southeastern Manitoba		Assembly yard B in Southeastern Manitoba		Assembly yard C in Southwestern Manitoba	
Sample #	Sample source	Cт	Source	Cт	Source	Cт
1	Waterer	25.3	Waterer/feeder	29.8	Loading Chute #2 bottom	31.3
2	Feeder	28.3	Waterer/feeder	28.8	Loading chute #2 Top	26.6
3	Loadout	24.7	Waterer/feeder	29.2	Pen 104	31.4
4	Hallway	27.0	Hallway	26.4	100 Centre alley sorting gate	33.1
5	Waterer	28.8	Waterer/feeder	31.2	Pen 203	33.8
6	60 Lbs Chain	0.0	Waterer/feeder	32.9	200 Alley	34.0
7	60 Lbs Feeder/waterer	33.9	floor	30.4	100 Alley	31.4
8	Feeder	29.4	Waterer/feeder	28.2	Pen 101	27.2
9	Hallway M	25.8	Floor-hall—	26.1	200 Alley Sorting Gate	31.3
10	Waterer	26.5	Floor receiving	27.8	S Scale Unload	0.0
11	Loadout M	24.6	Lower loadout	22.1	Pen WI	37.1
12	Feeder	33.9	Lower loadout	23.4	Pen 300	30.7
13	Loadout S	27.8	Receiving 4	32.5	Pen 96	31.8
14	Floor-pen	32.8	Scale	27.8	90 Alley	33.7
15	Waterer	33.2	Before scale	32.8	Blue Scale	35.2
16	Water/feeder	26.4	Receiving 3	33.2	Black Scale	31.0
17	Skid wet bucket	30.5	Cross over—	28.6	Pen 81	35.9
18	Feeder	27.2	Receiving 3 pen	26.3	Loading Dock 5	36.4
19	skids tire	31.3	Mid alley	31.4	Loading Dock 4 Top	32.1
20	scale	32.7	Waterer/feeder pen 4	25.3	Loading Dock 4 Bottom	33.7
21	Transition floor	33.8	Feeder/water	25.4	Unload Dock 3	37.0
22	Transition floor	33.9	Floor cross	28.0	Black Scale	36.7
23	Loading floor 1	0.0	Floor	23.3		
24	Feeder/waterer	0.0	After scale floor	24.7		
25	Waterers	36.0	Feeder/water	28.1		
26	Floor	33.5	Skid tires	33.3		
27	Loadout ramp	0.0	Water/feeder	27.8		
28	Alley way	36.3	Floor	28.2		
29	Feeder	0.0	Waterer/feeder	27.3		
30	Feeder/waterer	0.0	Thick tires	30.7		

Tissue scrapings in VDTM were transferred into 15 mL falcon tubes, centrifuged to pellet the tissue material which was then rinsed with Dulbecco’s PBS (D-PBS) to get rid of residual transport medium and emulsified to obtain a 10% weight/volume suspension in D-PBS. The suspension was clarified by centrifugation at 2000g for 20 min at 4°C and the supernatant treated with antibiotics for 30 min at room temperature.

### RNA extraction and RRT-PCR

RNA extraction from tissue suspensions and environmental samples was performed as previously described [17] using a MagMax-96 Viral RNA Isolation Kit, AM1836 (ThermoFisher Scientific Inc., USA) following manufacturer’s protocol. The MagMAX™ Express-96 Instrument and a Deep Well Magnetic Particle Processor (ThermoFisher Scientific Inc.) were used for RNA purification. SVA RNA was then detected by real-time reverse transcription polymerase chain reaction (RRT-PCR) that specifically amplifies a 117 bp region of the SVA 2C gene. The primers (SVA 4269- forward primer 5’- TCT CTT GCC CTA ACA CTG GGG—3’ and SVA reverse primer 5’- CTT GCC TCT AAG GAC CAC CACA- 3’) and the SVA Probe, 5’ 6FAM- TGG CCC AAA/ZEN^†^/GTC TCA CCA CTA TGA TCA ATG -IABkFQ^†^—3’ designed by Dr. Fabio Vannucci, University of Minnesota Veterinary Diagnostic Laboratory (unpublished) were custom made by IDT.

The AgPath® ID RT-PCR kit (AM1005, ThermoFisher Scientific Inc.) was used for SVA RRT-PCR. The template (5 μL) was added to a mastermix comprising 5.5μL of RNase-free water, 12.5 μL of 2x RT-PCR buffer, 1μL of 25x RT-PCR enzyme mix and 0.2 μM each of forward primer, reverse primer and probe in a final volume of 25 μL. The cycling parameters on ABI 7500 were: stage 1(10 min at 48°C), stage 2 (10 min at 95°C), stage 3 (10 sec at 95°C, 60 sec at 60°C repeated 40 times with collection of fluorescence). Crossing threshold (Ct) < 35.99 was considered positive for SVA genome.

### Virus isolation

Monolayers of a fetal porcine kidney cell line constitutively expressing α_v_β_6_ integrin (LFBKα_v_β_6_ cells, [18][19] were inoculated with tissue suspensions and environmental samples, the plates incubated for 2–3 days at 37° C and checked for cytopathic effect (CPE). If CPE was observed, supernatants were collected and tested for SVA by RRT-PCR.

### Genome amplification, sequencing and data analysis

Near complete genome of SVA was amplified directly from total RNA extracted as outlined above. Two overlapping RT-PCR runs were performed using SuperScript® One-Step RT-PCR System for Long Templates (Invitrogen) according to manufacturer’s protocol. Primers were designed from conserved regions according to available SVA sequences deposited in the GenBank. Primer sequences were as follows: set 1 (SVA-F59, 5’-AACCGGCTGTGTTTGCTAGAG-3’, SVA-R4176, 5’-ATAGTGGTGAGACTTTGGGCCAA-3’); set 2 (SVA-F3551, 5’-ATCTAGTCACTCTGGCCTCTC-3’, SVA-R7247, 5’-CCGACTGAGTTCTCCCAGAATC-3’).

The RT-PCR products were purified using a QIAquick Gel Extraction kit (Qiagen) and purified genomes were subsequently quantified using a Biodrop Touch UV spectrophotometer. Library construction was performed using the Ion Xpress™ Plus Fragment Library Kit (Life Technologies) utilizing a 5 minute shearing time. IonXpress™ Barcode adapters (Life Technologies) were applied to each full genome isolate. Sheared genomes were size selected with a PIPPIN-Prep using 2% agarose gels (Sage Science). Size-selected libraries were qualitatively assessed using the Agilent High Sensitivity DNA Kit and Agilent 2100 Bioanalyzer. A qPCR assay was performed on the ABI7500 Fast Real-Time PCR System (Applied Biosystems) and with the Ion Library Quantitation Kit (Life Technologies) to determine the template dilution factor required for emulsion PCR. Barcoded libraries were then pooled and DNA template prepared for sequencing using the Ion PGM^™^ Template OT2 Reactions 200 kit (Life Technologies) with the Ion OneTouch^™^2 System with ES for ion sphere particle (ISP) enrichment. Quality control of ISP’s was performed using an Ion Sphere^™^ Quality Control Kit and a Qubit fluorometer (Life Technologies). Sequencing was performed with an Ion Torrent PGM^™^ instrument using an Ion 314™ Chip Kit v2 (Life Technologies) and an Ion PGM^™^ Hi-Q^™^ Sequencing Kit. Whole genomes were assembled utilizing the DNAstar SeqMan NGen® software (Version 12.2.0; DNASTAR, Inc.). Pairwise nucleotide sequence alignments were performed using the Martinez-NW method) [20]and the Lipman-Pearson method [21]for protein alignments in MegAlign (Lasergene, version 12.2.0; DNASTAR, Inc.).

### Nucleotide sequences used in the study

All available full genome sequences of SVA were downloaded from GenBank on February 22, 2017. Sequences containing ambiguous bases were removed after initial alignment performed using Muscle in MEGA (version 7.0). Multiple sequence alignments were screened for recombinant sequences using the programs RDP, GENECONV, MAXCHI, CHIMAERA, 3SEQ, BOOTSCAN and SISCAN in the recombination detection program version 4 (RDP4) software package [22] using default settings. Potential recombinant sequences were identified when two or more methods were in agreement with p-values < 0.001. A total of 22 SVA sequences from GenBank were free of recombinant events when compiled with 11 sequences generated in this study. This led to a data set consisting of 33 SVA sequences analyzed in this report (Table 2).

**Table 2:** SVA strains used in phylogenetic analysis.

Strain	ID in network	Geographic region	Sample source	Collection date	Accession no.	Sequence reference
SVA/Canada/MB/NCFAD-104-1/2015	C104-1	Assembly yard A, Manitoba, Canada	Waterer	2015-10-30	KY486156	This study
SVA/Canada/MB/NCFAD-104-6/2015	C104-6	Assembly yard A, Manitoba, Canada	Chain	2015-10-30	KY486157	This study
SVA/Canada/MB/NCFAD-104-9/2015	C104-9	Assembly yard A, Manitoba, Canada	Hallway	2015-10-30	KY486158	This study
SVA/Canada/MB/NCFAD-108-12/2015	C108-12	Assembly yard B, Manitoba, Canada	Load out	2015-11-06	KY486159	This study
SVA/Canada/MB/NCFAD-108-16/2015	C108-16	Assembly yard B, Manitoba, Canada	Receiving area	2015-11-06	KY486160	This study
SVA/Canada/MB/NCFAD-108-20/2015	C108-20	Assembly yard B, Manitoba, Canada	Waterer/feeder	2015-11-06	KY486161	This study
SVA/Canada/MB/NCFAD-119-2/2015	C119-2	Assembly yard C, Manitoba, Canada	Loading chute	2015-12-01	KY486162	This study
SVA/Canada/MB/NCFAD-119-6/2015	C119-6	Assembly yard C, Manitoba Canada	Alley	2015-12-01	KY486163	This study
SVA/Canada/MB/NCFAD-119-7/2015	C119-7	Assembly yard C, Manitoba Canada	Alley	2015-12-01	KY486164	This study
SVA/Canada/ON/FMA-2015-0024T1/2015	C24T1	Ontario, Canada	Pig tissue biopsy	2015-10-10	KY486165	This study
SVA/Canada/ON/FMA-2015-0024T2/2015	C24T2	Ontario, Canada	Pig tissue biopsy	2015-10-10	KY486166	This study
SVA/USA/IA40380/2015	IA40380	Iowa, USA	Pig	2015-07-23	KT757280	Zhang et al. (2015)
SVA/USA/SD41901/2015	SD41901	South Dakota, USA	Pig	2015-07-31	KT757281	Zhang et al. (2015)
SVA/USA/IA46008/2015	IA46008	Iowa, USA	Pig	2015-08-25	KT757282	Zhang et al. (2015)
SVA/USA/MN15-84-4/2015	MN84-4	Minnesota, USA	Swab from mortality tractor bucket	2015-10-10	KU359210	Joshi et al. (2016)
SVA/USA/MN15-84-8/2015	MN84-8	Minnesota, USA	Mouse feces	2015-10-10	KU359211	Joshi et al. (2016)
SVA/USA/MN15-84-21/2015	MN84-21	Minnesota, USA	Swab from internal hallway	2015-10-10	KU359212	Joshi et al. (2016)
SVA/USA/MN15-84-22/2015	MN84-22	Minnesota, USA	Lesion swab from sow	2015-10-10	KU359213	Joshi et al. (2016)
SVA/USA/MN15-308-M32015	MN308	Minnesota, USA	Mouse small intestine	2015-10-10	KU359214	Joshi et al. (2016)
SVA/USA/KS15-01/2015	KS15-01	North Carolina, USA	Pig nasal swab	2015–06	KX019804	Chen et al. (2016)
SVA/USA/US-15-41901SD/2015	41901SD	South Dakota, USA	Pig vesicular lesion swab	2015-07-31	KU051394	Guo et al. (2016)
SVA/USA/US-15-40381IA/2015	40381	Iowa, USA	Pig vesicular lesion swab	2015-07-22	KU051393	Guo et al. (2016)
SVA/USA/US-15-40380IA/2015	40380	Iowa, USA	Pig vesicular lesion swab	2015-07-23	KU051392	Guo et al. (2016)
SVA/USA/US-15-39812IA/2015	39812IA	Iowa, USA	Pig vesicular lesion swab	2015-07-21	KU051391	Guo et al. (2016)
SVA/USA/GBI29/2015	GBI29	North Carolina, USA	Pig nasal/rectal swab	2015–08	KT827251	Hause et al. (2016)
SVA/USA/OH1/2015	OH1	Ohio, USA	Pig vesicle tab	2015-10-19	KU058182	Wang et al. (2016)
SVA/USA/OH2/2015	OH2	Ohio, USA	Pig vesicle tab	2015-10-19	KU058183	Wang et al. (2016)
SVA/BRA/MG1/2015	BRAMG1	Minas Gerais, Brazil	Pig vesicular fluid	2015-02-01	KR063107	Vannucci et al. (2016)
SVA/BRA/MG2/2015	BRAMG2	Minas Gerais, Brazil	Pig vesicular fluid	2015-02-01	KR063108	Vannucci et al. (2016)
SVA/BRA/G03/2015	BRAG03	Goias, Brazil	Pig vesicular fluid	2015-02-01	KR063109	Vannucci et al. (2016)
SVA/China/CH-01-2015/2015	CH01	Guangdong, China	Pig	2015–05	KT321458	Wu et al. (2016)
SVA/China/CH-LX-01-2016/2016	CHLX01	China	Pig	2016–01	KX751945	Zhao et al. (2017)
SVA/China/HB-CH-2016/2016	HBCH	Hubei, China	Pig vesicular lesion tissue	2016–03	KX377924	Qian et al. (2016)

### Phylogenetic analyses

Rate of nucleotide substitution per site per year and the time to the most recent common ancestor (TMRCA) of 33 SVA strains were estimated from polyprotein coding region by the Bayesian Markov Chain Monte Carlo (BMCMC) method using the program BEAST, version 2.3.0 [23]. The best fit nucleotide substitution model, TN93 + G in the MEGA 7.0.18 software [24], was used. The age of the viruses was defined as the date of sample collection. The relaxed uncorrelated exponential clock and exponential population size model were found to be the best fit to the data. These models were compared to a strict molecular clock and a constant-size population coalescent model. Model selection was performed by comparing the model marginal log-likelihood through the Akaike’s information criterion [25]. For the dataset, at least two independent BEAST analyses were run for a sufficiently long time to ensure that all parameters had an effective sample size (ESS) of > 200. Convergences and ESS of the estimates were checked using Tracer v1.6 [26]. A maximum clade credibility (MCC) phylogenetic tree was generated to summarize all 10,000 trees after a 10% burn-in using TreeAnnotator in BEAST [23]. The time-stamped phylogenetic tree was visualized and annotated using FigTree v1.4.2 [26]. Maximum likelihood phylogenetic analysis, using MEGA 7.0.18 software, was also carried out on the same data set in order to compare with the Bayesian method. To assess the robustness of different nodes, bootstrap analysis was undertaken using 1000 replicates of the data set. The polyprotein gene alignment was used to construct a phylogenetic network using the Median Joining method in the program Network v5.0 (http//www.fluxus-engineering.com).

### Analysis of selection pressure

Site-specific selection pressures for polyprotein sequences of 22 SVA strains were measured as nonsynonymous (dN) -synonymous (dS) nucleotide substitutions per site. In all cases, the difference were estimated using the single-likelihood ancestor counting (SLAC), fixed-effects likelihood (FEL), internal fixed-effects likelihood (IFEL), and random effects likelihood (REL) methods [27,28] available in Datamonkey [28,29] online version of the HyPhy package [28]. All analyses utilized the TN93 + G nucleotide substitution model, which was tested as the best fitting model for the data set, and employed input Neighbor Joining phylogenetic trees. A cut-off p-value to classify a site as positively or negatively selected was set at 0.01 for SLAC, FEL, and IFEL methods. The cut-off value for the Bayes factor in the REL method was set at 100 to reflect a positive or negative selection at a given site.

### 3D structure manipulation

Protomer structure of prototype SVA (PDB # 3CJI; [30] was manipulated with the UCSF Chimera package from the Resource for Biocomputing, Visualization, and Informatics at the University of California, San Francisco [31], supported by NIH P41 RR-01081). Resulting image was imported into Adobe Photoshop and assembled with Adobe Illustrator (Adobe).

## Results

### Real-time RT-PCR

All samples from Manitoba farms were negative for SVA. Two tissue scrapings collected from pigs in Ontario were positive for SVA with Ct values of 14. Environmental samples from assembly yards A, B and C in Manitoba were 73.3%, 100% and 81.8% positive for SVA respectively with Ct values of 22 to 35 (Table 1).

### Virus isolation

Cytopathic effect was observed in LFBK cells inoculated with tissues suspensions from each of the 2 tissue scrapings collected from pigs in Ontario. Virus was isolated from selected SVA genome-positive environmental samples after 48h incubation on LFBK cells. Virus was also isolated from an environmental sample (NCFAD-104 #6) initially negative by RRT-PCR. These isolates were confirmed as SVA by RRT-PCR.

### Sequence analysis

**Fig 1:**
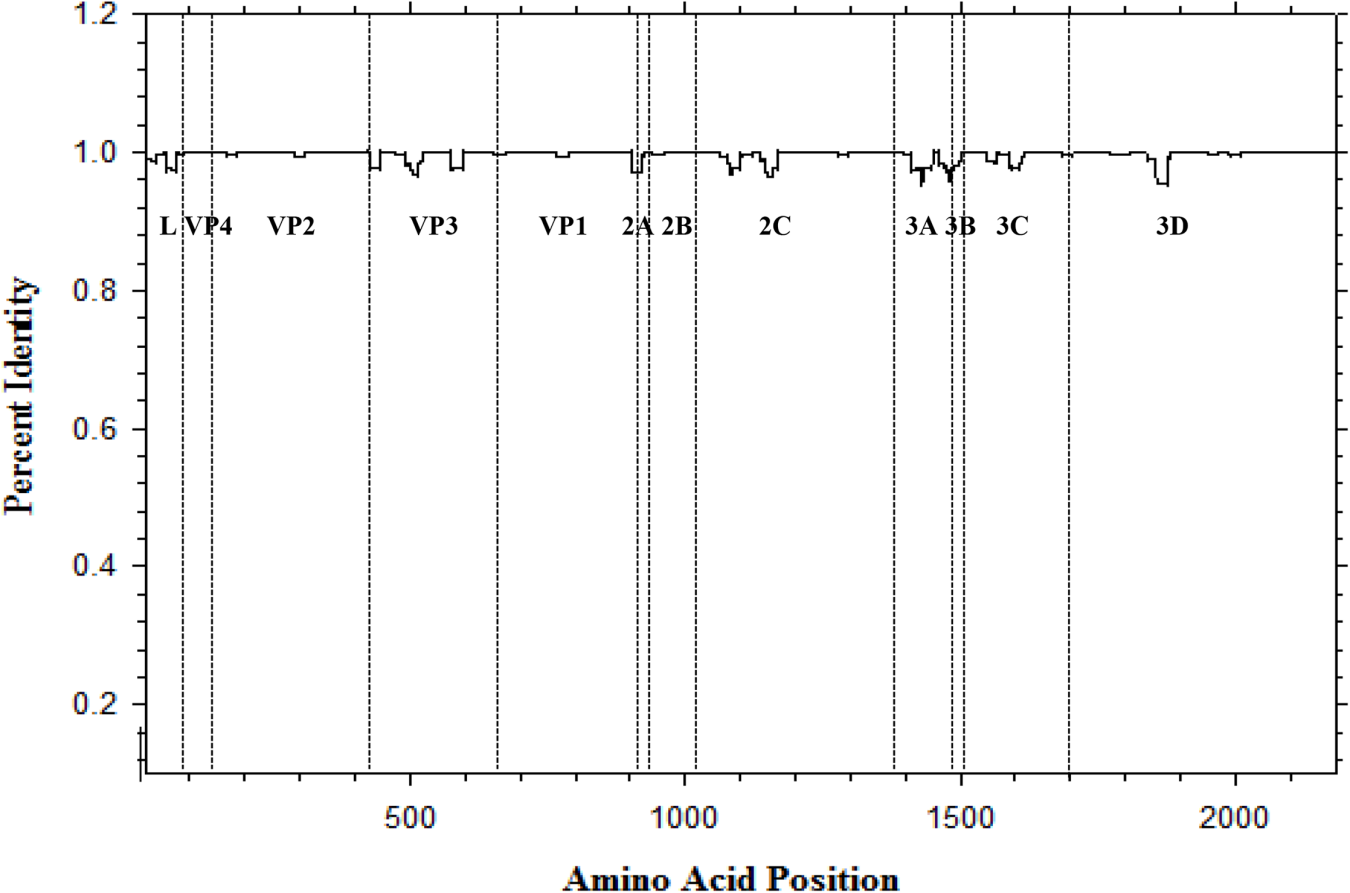
Window-averaged scores for sequence identity among polyproteins of the 33 SVA strains. Identity scores are averaged over running windows of 21 amino acids and centered at consecutive amino acid positions. The variable regions are indicated by their represented mature proteins.

The nucleotide sequence coding for complete polyprotein was determined to contain 6543 nucleotides for each of the 11 Canadian SVA strains. There were no gaps relative to one another when aligned. The 11 Canadian SVA polyproteins were each deduced to be 2181 amino acids long with no insertions or deletions relative to each other. The polyprotein is processed to 12 mature proteins consisting of L, VP4, VP2, VP3, VP1, 2A, 2B, 2C, 3A, 3B, 3C, and 3D by virus-encoded proteinases [1]. Pairwise sequence comparisons of the 12 protein coding regions among the 11 Canadian SVA strains showed identity of 97–100% at both nucleotide and amino acid levels. No amino acid sequence differences were found in VP4, 2A, 2B, and 3B regions in the alignment of the 11 Canadian strains. Nucleotide and amino acid identities ranged from 92.4% to100% and 93.3% to 100%, respectively, when the 11 Canadian SVA strains were compared with 22 SVA strains from Brazil, China and the United States with respect to 12 mature protein coding regions. Out of 12 protein coding regions compared, two proteins (VP4 and 3B) were found to have no mismatches in the amino acid alignment of the 33 SVA strains. Weighted window-averaged analyses of 33 SVA polyprotein identities revealed high overall identity, with only a few regions where small variations were observed (Fig 1). These amino acid substitutions were located in the L, VP2, VP3, VP1, 2C, 3A, 3C, and 3D regions. Out of a total of 650 nucleotide substitution sites identified in the polyprotein region, mutations were defined as follows: 66 nonsynonymous (21 singletons) and 584 synonymous.

The nucleotide and amino acid differences from averaging over all sequence pairs between country groups are shown in Table 3. The nucleotide differences are ~ 3%, whereas amino acid differences are ~1% between country groups. Although the significance of percentage differences in nucleotide sequence comparisons is not clearly understood, a value of approximately 15% nucleotide sequence difference is used to differentiate major genotypes of picornaviruses [32,33].

**Table 3:** Evolutionary divergence over SVA sequence pairs between countries.

Country	Canada	USA	Brazil	China
Canada		0.8	0.9	0.7
USA	3.1		0.7	1.1
Brazil	2.8	2.2		1.2
China	2.8	3.4	3.2	

Percent amino acid differences indicated in upper triangle and percent nucleotide differences in lower triangle.

**Fig 2:**
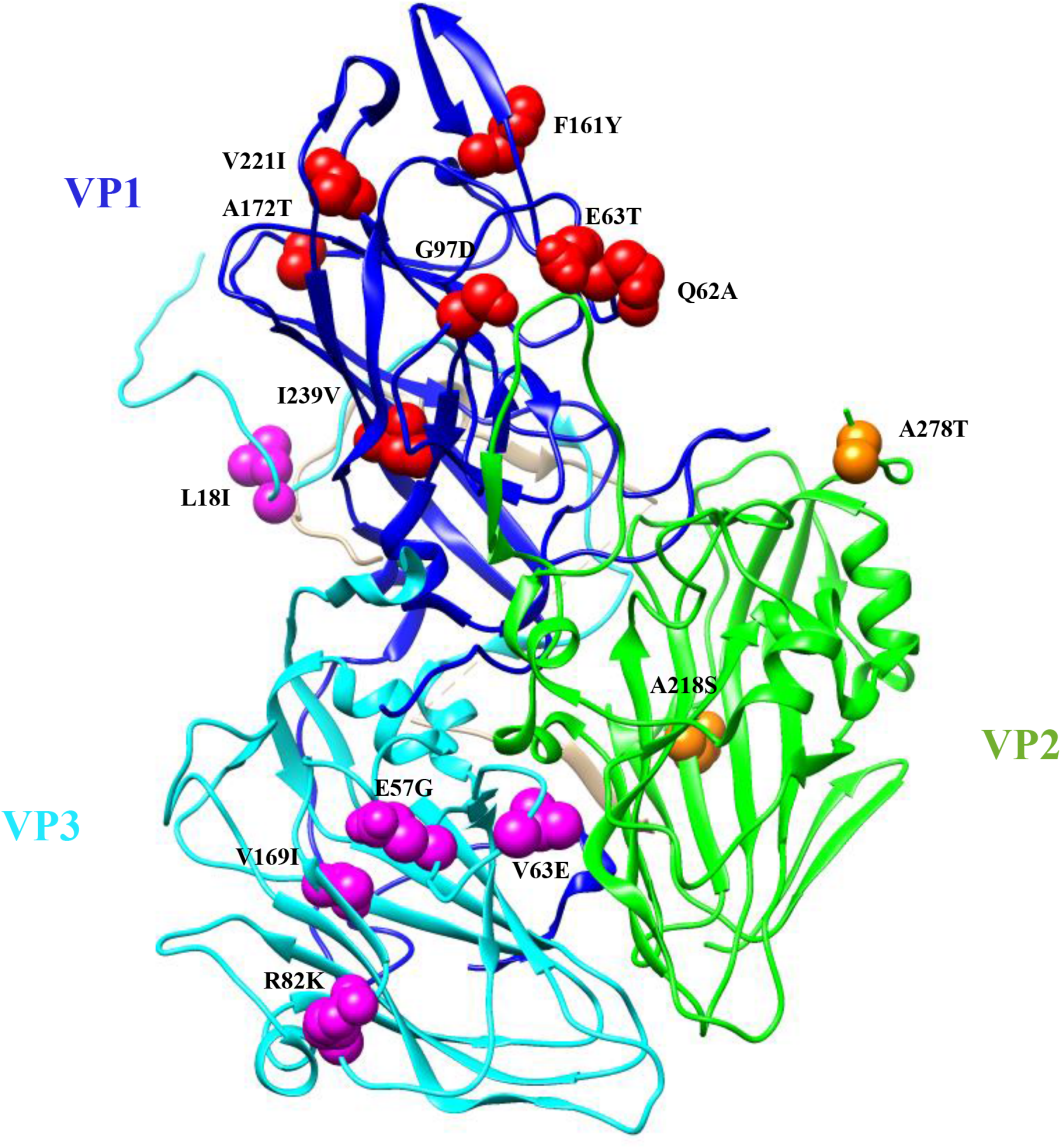
Localization of nonsynonymous mutations detected in all 33 SVA strains in comparison to prototype SVV-001 and a positively selected residue by REL method in the protomer structure of SVV-001. The structure was manipulated with Chimera®. VP1, VP2, VP3 and VP4 subunits are shown in blue, green, cyan and light gray, respectively. Mutated amino acids are shown in spheres.

The polyprotein sequences of the 33 SVA strains were compared with the prototype strain SVV-001 (accession no. DQ641257), which was isolated in the USA in 2002. A total of 25 non-synonymous mutations distributed in 8 mature virus proteins (VP2, VP3, VP1, 2B, 2C, 3A, 3C, and 3D) were identified in all 33 SVA strains (Table 4). Fourteen of these substitutions were present in the VP1, VP2, and VP3, from which 7 were surface- exposed. These mutations in the structural proteins were annotated in the 3D structure of the SVV-001 (Fig 2).

**Table 4:** Non-synonymous mutations detected in all 33 SVA strains compared to prototype strain SVV-001.

VP2	VP3	VP1	2B	2C	3A	3C	3D
N218S	L18I	Q62A	D42N	N292S	S19T	I81V	I9V
A278T	E57G	E63T	V57I	T304A	T31A	I81L	V131A
	V63E	G97D			G82E	S120A	
	T77A	F161Y			S83P	E141D	
	R82K	A172T					
	V169I	V221I					
		I239V					

### Evolutionary rate and origin

Rate of nucleotide substitution and TMRCA of the 33 SVA strains were estimated for the polyprotein coding region using a BMCMC method in BEAST [23]. The relaxed uncorrelated exponential clock and exponential population size model were selected based on marginal log likelihood. The analyses showed that the mean substitution rate was 9.96×10^−3^ substitutions per site per year (s/s/y) with a 95% highest posterior density (HPD) interval of 4.75×10−3–1.59×10^−2^ s/s/y. TMRCA estimation showed that the origin of the 33-SVA dataset dated back to mid-June 2013 (95% HPD: mid-June 2011 –late September 2014). The common ancestors of SVA from South Eastern and Central Brazil, the United States, China, and Canada within the dataset were estimated to be late October 2014, early July 2014, early March 2015, and late January 2015, respectively.

### Phylogenetic analyses

**Fig 3:**
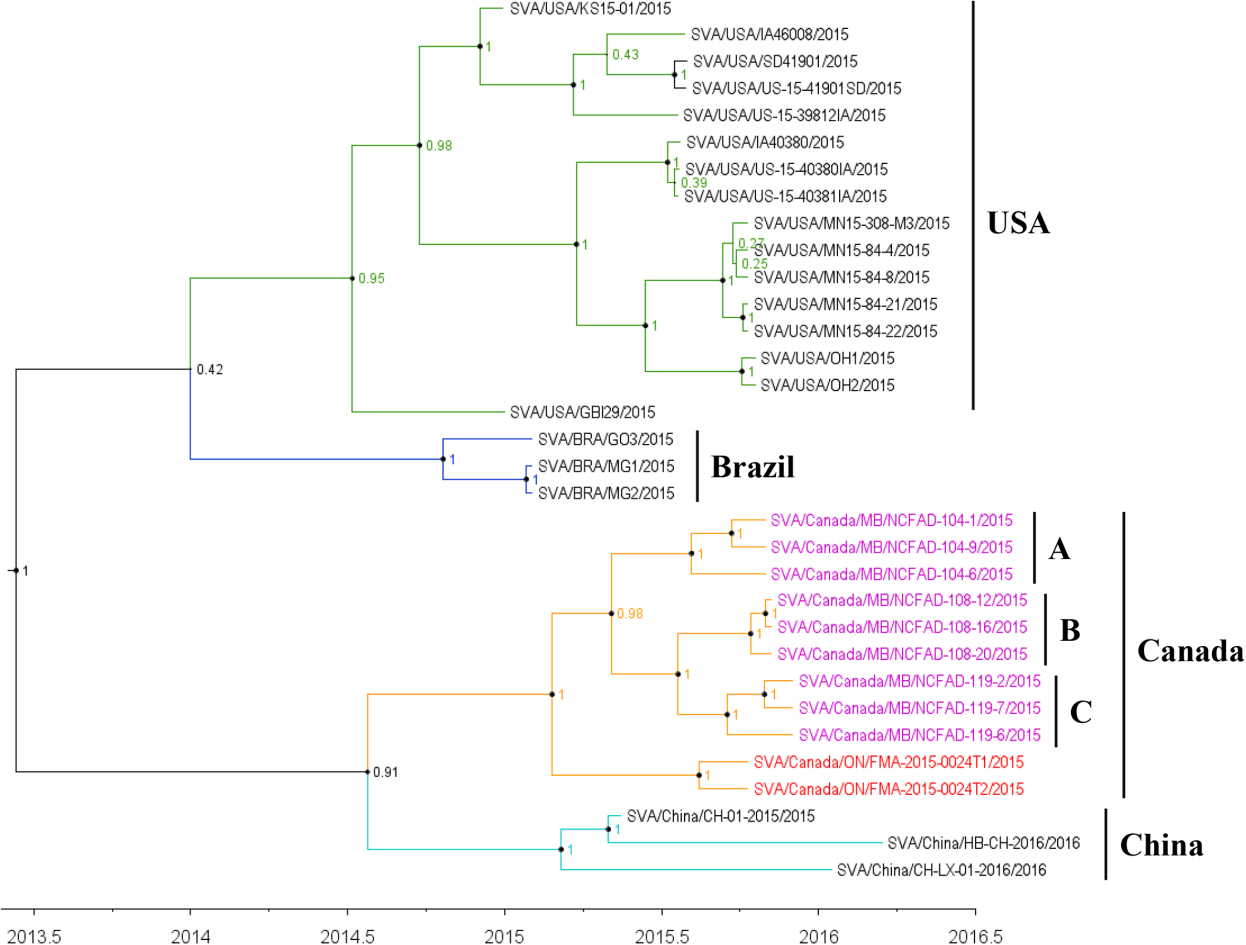
Time-scaled phylogenetic tree inferred using Bayesian MCMC analysis for the polyprotein coding sequences of 33 SVA strains. Branches are coloured according to geographic origin of the country. Strains from Manitoba are highlighted in magenta and from Ontario in cyan. Nodes supported by ≥ 0.7 posterior probability are indicated by the solid circles and sized by posterior probability. A, B, and C are sequences from assembly yards A, B, and C, respectively.

**Fig 4:**
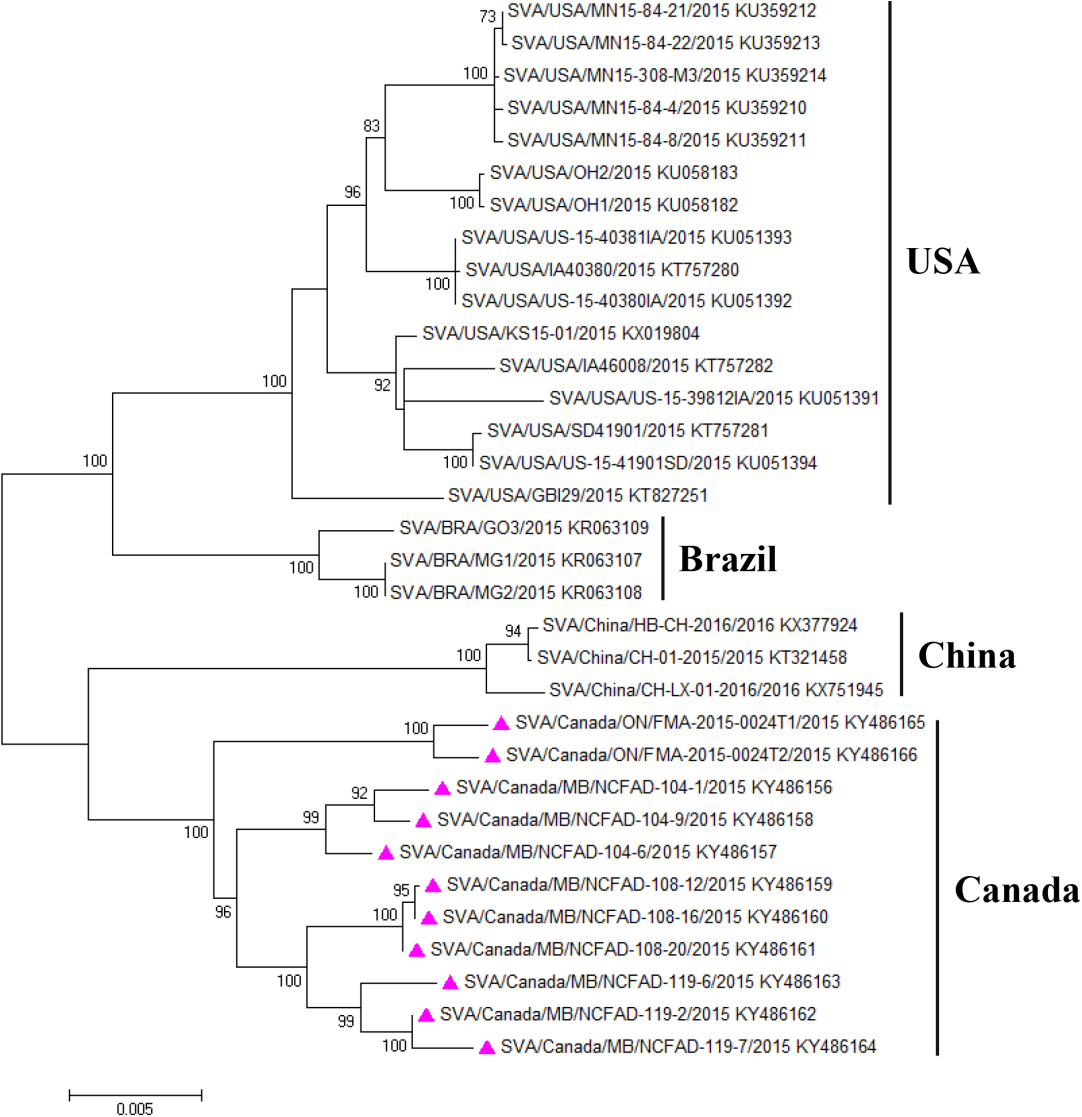
Maximum likelihood tree of the polyprotein coding sequences of 33 SVA strains. The numbers at nodes represent bootstrap values (>70%), while branch lengths are scaled according to the numbers of nucleotide substitutions per site. The tree was mid-point rooted.

**Fig 5:**
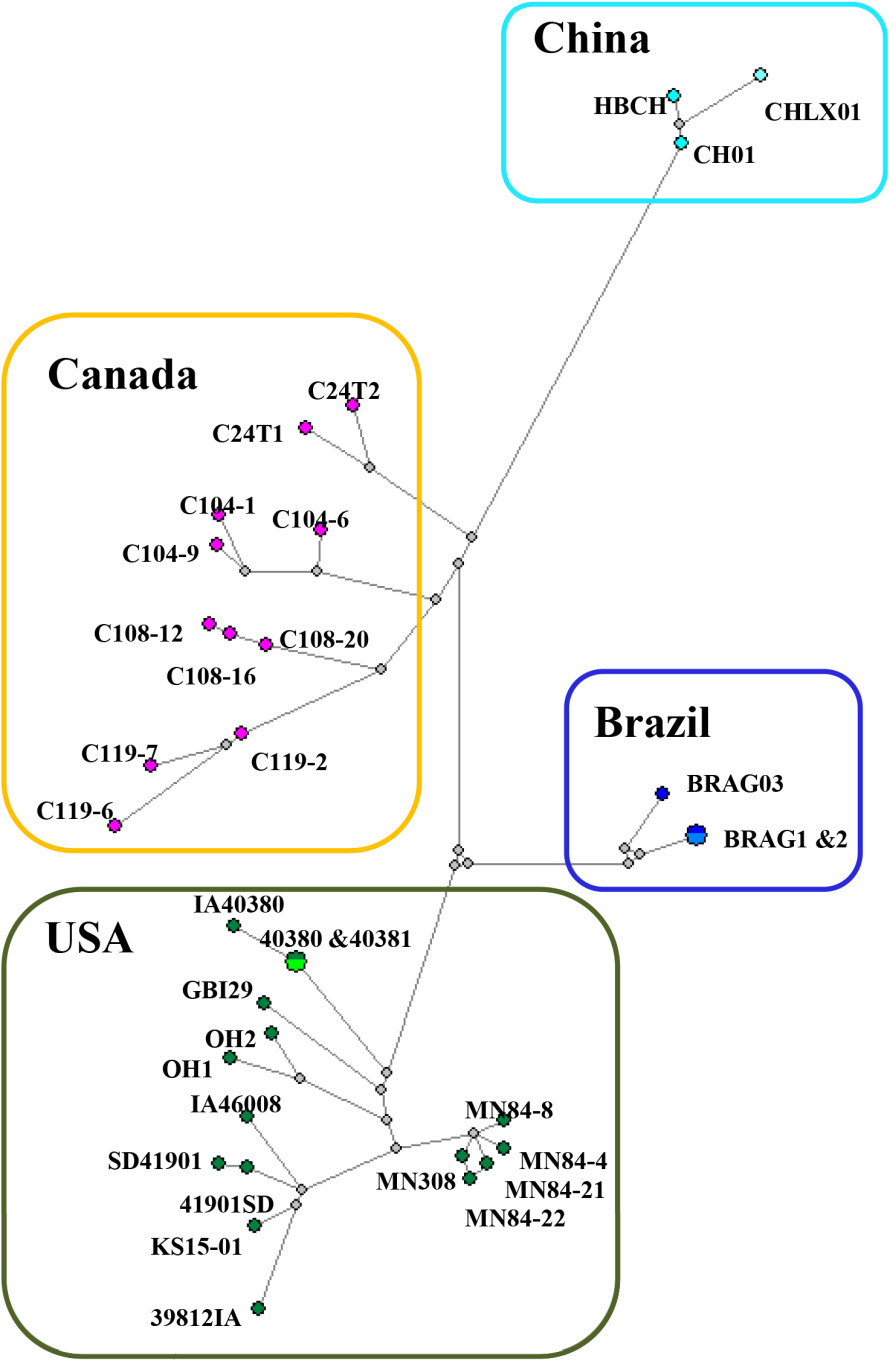
Median-joining phylogenetic network of 33 SVA strains constructed from an alignment of polyprotein coding sequences. This network includes all of the most parsimonious trees linking the sequences. Each unique sequence is represented by a coloured circle sized relative to its frequency in the dataset. Branch length is proportional to the number of mutations.

Phylogenetic trees of 33 SVA sequences were constructed from polyprotein coding sequences using Bayesian inference (Fig 3 and Maximum Likelihood (ML) methods (Fig 4). The topology of the MCC tree was similar to that inferred using ML method. Four genetic clusters, which fall into 4 geographically distinct regions (USA, Brazil, Canada, and China), were shown in phylogenetic trees that were well supported statistically. Three sub-clusters were inferred within Canadian cluster corresponding to assembly yards A, B, and C, respectively (Fig 3). A Median Joining phylogenetic network using the polyprotein nucleotide sequence alignment with the program NETWORK (v5.0) was constructed to infer transmission network (Fig 5). The network showed that virus sequences were grouped in multiple clusters, including the 4 major clusters identified with the Bayesian and ML methods. The four major clusters were connected at the base of the network by multiple calculated ancestors, reflecting four different geographic locations of the country (Fig 5). Sequences within these 4 clusters were separated in average by 18–74 nucleotide (4–7 amino acids) differences, whereas 143–215 nucleotide (15–26 amino acids) 34 differences were observed between clusters. Within each of the four major clusters (Canadian cluster, Brazilian cluster, Chinese cluster, and U. S. cluster) the sequences were grouped corresponding to their sampling locations. Notably, the network showed that strain C119-2 was the closest ancestor to a group of 2 samples (C119-7 and C119-6; Fig 5). Also, according to the network, strain C108-20 was the earliest ancestor of the strain C108-16, whereas strain C108-12 was the descendent of strain C108-16.

### Selection pressure acting on polyprotein coding region

We assessed the selection pressure acting on the polyprotein coding region using the HyPhy package in Datamonkey [28]. The SLAC, FEL, IFEL, REL methods were used to identify sites under positive selection. The analysis revealed that the vast majority of codons were subject to purifying selection. All methods failed to detect positive selection in any of the protein coding regions under the significance levels described in the materials and methods.

## Discussion

In this study we analysed SVA evolutionary potentials from 33 genomes sampled in Canada, the United States, China, and Brazil over 12 months of divergence time in 2015/2016. Results obtained in this study showed that polyprotein coding region was diversified between samples of different countries, resulting in four distinct clusters observed in the phylogenetic trees. The estimated substitution rate for the polyprotein coding region was 9.96 ×10^−3^ substitutions per site per year, which is among the highest observed for picornaviruses [34]. It suggests that SVA genetic diversity can be produced within a short period of time.

Analysis of selection pressure showed that this rapid evolutionary rate was mainly driven by purifying selection pressure. TMRCA estimation indicates that the origin of the recent SVA cases dated back to mid-June 2013 with common ancestors dating to late October 2014 and early July 2014 for Brazil and USA, respectively. This correlates with the fact that since November 2014, SVA was frequently reported in swine in Brazil, with increased numbers being reported in 2015 [4, 5]. Several outbreaks of SVA in pigs were reported in the summer of 2015 in the United States but SVA may have already been present a year early before it was detected during the outbreaks. The common ancestor for Canadian SVA strains was estimated to be late January 2015, suggesting that the SVA may have been introduced into assembly yards in Manitoba or Ontario months before SVA was detected from infected sows in October. We did not observe identical virus sequences within or between assembly yards, indicating that the SVA is diversifying and each assembly yard may contain a wide variety of closely related virus variants (confirmed by our deep sequencing analysis, data not shown) with variable fitness. The transmission network showed that no direct links were identified between assembly yards, suggesting that point source introductions may have occurred. The detection and isolation of SVA from various environmental samples in this study are suggestive of broad areas of contamination with SVA although the mechanisms of virus dissemination have to be determined. However, it is likely that animal movement within each assembly yard may play a role in the spreading of the virus according to our phylogenetic transmission network analysis.

Our data showed that 90% (584 out of 650) of nucleotide substitutions from 33 SVA strains were synonymous, which is consistent with strong negative selection drawn from our selection pressure analysis. Indeed, purifying selection is common in RNA virus evolution. Previous reports have shown that 73% of nucleotide substitutions from 46 RNA viruses were synonymous [35]. The evolutionary “hot spots” from 66 nonsynonymous mutations identified in the polyprotein region were dispersed in L, VP2, VP3, VP1, 2C, 3A, 3C, and 3D regions. These amino acid substitutions may confer advantages that increase the fitness of the virus in the host. Comparisons of 33 SVA strains with the prototype strain SVV-001 yielded 25 non-synonymous mutations in the polyprotein region for all strains, suggesting that theses substitutions have been fixed in the population during evolution over the past decade. Despite undetermined biological functions on these substituted residues, it is possible that some surface-exposed residues in the structural proteins VP1, VP2, and VP3 of the virus are antigenic sites and changes on these residues may disguise them from being recognized by the immune system. We previously generated a panel of monoclonal antibodies (mAb) against SVV-001 [7]. One of the mAbs characterized as a neutralizing mAb has failed to recognise recent strains of SVA, suggesting mutations in the binding site of the mAb (unpublished observation). Determination of the epitope of this mAb could yield valuable insight into possible immune evasion mechanisms of SVA. Interestingly, in our previous study [7], inoculation of pigs with SVV-001 did not cause any clinical symptoms associated with the infection, whereas in the study by Montiel et al and Joshi et al [8][9], inoculation of pigs with the current strains of SVA led to vesicular lesions. Taken together, the fixed substitutions observed from comparisons with prototype SVV-001 d are likely associated with SVA adaptation and increased pathogenicity in current SVA strains.

Overall, our results suggest that a combination of evolutionary processes such as multiple mutations at variable sites and purifying selection drove the genetic diversity observed in the current SVA strains. Future studies combining genetic data with epidemiological data should provide a better resolution of the phylogenetic transmission network and further understanding of the mechanisms involved in SVA spread during an outbreak.
